# Editorial: Physical and psychological proximity in humans: From the body to the mind and vice-versa

**DOI:** 10.3389/fpsyg.2023.1113851

**Published:** 2023-02-02

**Authors:** Chiara Fini, Dimitris Bolis, Quentin Moreau, Vanessa Era

**Affiliations:** ^1^Department of Dynamic and Clinical Psychology and Health Studies, Sapienza University of Rome, Rome, Italy; ^2^Independent Max Planck Research Group for Social Neuroscience, Max Planck Institute of Psychiatry, Munich, Germany; ^3^Department of System Neuroscience, National Institute for Physiological Sciences (NIPS), Okazaki, Japan; ^4^Precision Psychiatry and Social Physiology Laboratory (PPSP), CHU Sainte-Justine Research Center, Montreal, QC, Canada; ^5^Department of Psychiatry, University of Montréal, Montreal, QC, Canada; ^6^Department of Psychology, Sapienza University, Rome, Italy; ^7^IRCCS Fondazione Santa Lucia, Rome, Italy; ^8^Fondazione Istituto Italiano Di Tecnologia (IIT), Sapienza University of Rome and Center for Life Nano- & Neuroscience, Rome, Italy

**Keywords:** internal bodily signals, self-consciousness, human-human interaction, stress responses, interpersonal proximity

There is now a large consensus that mental processes are embodied phenomena (e.g., Clark, 1999; Barsalou, 2008; Adams, 2010; Shapiro, 2014; Da Rold, 2018; Zwaan, 2021; Ale et al., 2022). In the field of Embodied Cognition, the relationship between bodily, cognitive, and emotional states has been under intense investigation (e.g., Wilson, 2002; Shapiro, 2010; Costantini et al., 2011; Winkielman et al., 2015; Baumeister et al., 2017; Fini et al., 2017, 2021a). In the last years, specific attention has been given to interoceptive signals i.e., the signals coming from the inner parts of the body, such as heartbeats, breaths, gastrointestinal signals, and other internal sensations (e.g., Tsakiris et al., 2011; Pezzulo et al., 2018; Porciello et al., 2018; Iodice et al., 2019; Tschantz et al., 2022). These signals are important to assess internal emotional states (Critchley and Garfinkel, 2017), defining self-boundaries (Monti et al., 2021, 2022), adaptively regulating our physical needs (Pezzulo et al., 2015) and contributing also to the expression and maintenance of psychological disorders (Paulus and Stein, 2010). Although the understanding of the impact of bodily signals on psychological functioning has significantly enriched our knowledge in the field, additional research has yet to be conveyed to explore the intimate link between interoceptive signals and mental life. With the current research theme, we collected a number of diverse contributions, illustrating how internal bodily signals inform our mental processes: ranging from functions pertaining the bodily self, word processing, and stress management, to (interlinked) complex interpersonal dynamics. An integration of such diverse research strands, we believe, will be critical for the understanding of complex psychological and social processes, lying at the heart of what makes us humans (e.g., decisions, emotions, interpersonal coordination). By recognizing and detecting signals of different nature—bodily, psychological and interpersonal—this research field adopts a holistic approach to the study of human behavior.

One of the most popular experimental paradigms that allows the study of the contribution from both interoceptive and exteroceptive processing (e.g., vision, hearing) to self-awareness is the rubber hand illusion (RHI). By stroking a fake, rubber hand while synchronously stroking a participant's hidden hand, an illusion that the rubber hand belongs to oneself is induced and participants report that the location of their real hand is closer to the rubber hand than it really is (Botvinick and Cohen, 1998).

On that account, Della Longa et al. demonstrated that the RHI equally emerges with pleasant and painful stimulations. Their results confirm the importance of interoceptive signals (affective touch and painful stimulations compared to neutral touch) in promoting self-other distinction and bodily ownership.

The role of interoceptive signals in human cognitive functioning does not end with self-awareness. Instead, it also taps into other cognitive mental processes, such as language acquisition and conceptual processing. Paoletti et al. showed that toddlers engaged in a lexical decision task with abstract and concrete concepts presented an increase in temperature of the nasal tip, possibly indicating an increased parasympathetic dominance during the processing of abstract concepts. As the Words as Social Tools (WAT) Theory (Borghi et al., 2017, 2018, 2019) claims that abstract concepts are socially grounded, they might trigger a socio-linguistic component which might be more likely expressed by the predominance of the parasympathetic over the sympathetic system. Thus, the bodily temperature pattern, which signals a specific autonomic nervous system activation, seems to be associated with higher-level cognitive functions such as linguistic processing.

Internal bodily states are also sensitive to stressful contextual conditions. Therefore, various bodily signals are often used as parameters to probe the physiological status of the organism immersed in different environments. In their article, Peters et al., redefined different types of stress by specifying their internal bodily correlates. They identified “good stress”, “tolerable stress”, and “toxic stress”. In “good stress” humans adopt the most adequate response to face the stressful stimulus, in order to reduce the uncertainty or environmental “entropy” [for the notion of Free Energy Principle see Friston et al. (2006) and Friston (2010)] and it is usually associated with cerebral insulin suppression, brain-induced ketogenesis, and brain-induced heart rate acceleration. The “tolerable stress”, consisting of the habituation to stressful situations by flattening/broadening our own goals, leads to a reduction of arousal and hormonal responses. Finally, in the case of “toxic stress”, people manifest a permanent brain energy-consuming arousal state during the day and the lack of brain energy-saving deep sleep at night which contribute to the overall increase in total brain energy consumption. In particular, the authors suggested how some physiological conditions caused by a maladaptation to chronic stress might be at the origin of metabolic dysregulation, such as Type 2 diabetes mellitus and obesity. Thus, internal bodily signals appear to also reflect how humans interact with the environment.

The environment can be perceived as more or less stressful and aversive across time and context. One prominent example is social proximity before and during the COVID-19 situation. Before the pandemic, being physically closer to friends and family was often considered a source of pleasure and reassurance, whereas during peaks of infection, it was a reason for anxiety and distress. Scerrati et al., provided evidence for the flexibility of categorization processing depending on social proximity evaluation (see also Fini et al., 2021b). In detail, they showed, through an affective priming paradigm, that those who evaluated social proximity as positive processed positive words preceded by images of social proximity faster, while the contrary applied to individuals evaluating social proximity as negative. Although interoceptive signals were not directly measured, these results highlight how an internal emotional state affects the perception of socially relevant information.

Until now we underlined the entanglement among internal bodily signals, bodily awareness, cognitive processes, human-environment interaction, and the processing of socially relevant information. Ultimately, it is worth mentioning the impact of interoceptive signals on human-human interactions. Indeed, the autonomic nervous system may play a crucial role also in regulating individuals' ability to coordinate and adapt to others' behavior (Thayer et al., 2009) during interpersonal interactions. In these contexts, where two or more individuals coordinate their actions, thoughts, or words, and continuously predict, monitor, and adapt to each other's behavior (e.g., Vesper et al., 2010; Hale et al., 2020; Moreau et al., 2020, 2022; Boukarras et al., 2021, 2022; Boukarras et al.; Era et al., 2022; Sacheli et al., 2022), the study of collective psychophysiology has gained attention — especially from a second-person perspective (cf. Schilbach et al., 2013; Bolis et al., 2023). Research on this phenomenon, conceptualized as the relationship between pairs or groups of individuals' physiological dynamics, has shown how interacting with others results in interpersonal synchrony in cardiac (Danyluck and Page-Gould, 2019), electrodermal (Zeevi et al., 2022) and respiratory (Müller and Lindenberger, 2011) signals. More importantly, interpersonal attunement (e.g., coordination, synchrony, or similarity) appears to be associated with the quality of the interactions (Palumbo et al., 2017; Bolis et al., 2021; Czeszumski et al., 2022). In this regard, Boukarras et al., shed light on the importance of capturing interpersonal synergies and dynamics, also indexed by the relationship between two or more individuals' internal bodily signals, when investigating working environments (i.e. Organizational Neuroscience).

Taken together, the above-mentioned studies point to the importance of exploring internal bodily processing in tight interrelation with the subjective and intersubjective dimensions of human behavior, cognitive functioning, and social interaction, across scales and contexts, ranging from the simplest processes of self-awareness to the most complex social structures. A challenging empirical research that remains largely unexplored concerns the investigation of collective psychophysiology during so-called high-level psychological functions, e.g., internalizing and processing complex abstractions, especially within real-time social interactions (cf. Bolis and Schilbach, 2020; Borghi and Fernyhough, 2023; Fini et al., 2023).

## Author contributions

CF and VE conceived and wrote the manuscript. DB and QM critically revised the manuscript. All authors approved the final version of the manuscript.
